# *Leptospira Interrogans* Induces Fibrosis in the Mouse Kidney through Inos-Dependent, TLR- and NLR-Independent Signaling Pathways

**DOI:** 10.1371/journal.pntd.0002664

**Published:** 2014-01-30

**Authors:** Martine Fanton d'Andon, Nathalie Quellard, Béatrice Fernandez, Gwenn Ratet, Sonia Lacroix-Lamandé, Alain Vandewalle, Ivo G. Boneca, Jean-Michel Goujon, Catherine Werts

**Affiliations:** 1 Institut Pasteur, Biology and Genetics of the Bacterial Cell Wall Unit, Paris, France; 2 INSERM, équipe Avenir, Paris, France; 3 Service d'Anatomie et Cytologie Pathologiques, CHU Poitiers; Université de Poitiers, Poitiers, France; 4 Institut National de la Recherche Agronomique, Infectiologie et Santé Publique, Nouzilly, France; 5 INSERM U773 and Université Paris 7 - Denis Diderot, Paris, France; Universidad Peruana Cayetano Heredia, Peru

## Abstract

**Background:**

*Leptospira (L.) interrogans* are bacteria responsible for a worldwide reemerging zoonosis. Rodents carry *L. interrogans* asymptomatically in their kidneys and excrete bacteria in the urine, contaminating the environment. Humans get infected through skin contact and develop a mild or severe leptospirosis that may lead to renal failure and fibrosis. *L. interrogans* provoke an interstitial nephritis, but the induction of fibrosis caused by *L. interrogans* has not been studied in murine models. Innate immune receptors from the TLR and NLR families have recently been shown to play a role in the development and progression of tissue fibrosis in the lung, liver and kidneys under different pathophysiological situations. We recently showed that TLR2, TLR4, and NLRP3 receptors were crucial in the defense against leptospirosis. Moreover, infection of a human cell line with *L. interrogans* was shown to induce TLR2-dependent production of fibronectin, a component of the extracellular matrix. Therefore, we thought to assess the presence of renal fibrosis in *L. interrogans* infected mice and to analyze the contribution of some innate immune pathways in this process.

**Methodology/principal findings:**

Here, we characterized by immunohistochemical studies and quantitative real-time PCR, a model of *Leptospira*-infected C57BL/6J mice, with chronic carriage of *L. interrogans* inducing mild renal fibrosis. Using various strains of transgenic mice, we determined that the renal infiltrates of T cells and, unexpectedly, TLR and NLR receptors, are not required to generate *Leptospira*-induced renal fibrosis. We also show that the iNOS enzyme, known to play a role in *Leptospira*-induced interstitial nephritis, also plays a role in the induction of renal fibrosis.

**Conclusion/significance:**

To our knowledge, this work provides the first experimental murine model of sustained renal fibrosis induced by a chronic bacterial infection that may be peculiar, since it does not rely on TLR or NLR receptors. This model may prove useful to test future therapeutic strategies to combat *Leptospira*-induced renal lesions.

## Author Summary

Leptospirosis is a bacterial disease transmitted by asymptomatic rodents to humans. The symptoms may be mild, or severe with kidney failure. Renal fibrosis, occurring during inflammatory situations, is characterized by the pathological accumulation of extra-cellular matrix components and can compromise the kidney functions of patients with leptospirosis. Recent research revealed that both innate and adaptive immune responses are involved in the establishment of fibrosis, in several organs and in different pathophysiological situations. In the present study, we characterized a mouse model of chronic infection with *Leptospira* that provokes mild renal fibrosis. We show that fibrogenesis requires the presence of live *Leptospira* in the kidney and that B and T cells from the adaptive immune response do not participate in the induction of renal fibrosis. Unexpectedly, we also found that innate immune receptors, TLRs and NLRs, are not involved in the *Leptospira*-induced fibrosis. Finally, we show that the enzyme responsible for NO production, iNOS, known to participate in renal inflammatory lesions induced by *Leptospira*, is also involved in renal fibrosis. Our work provides a novel mouse model to study fibrosis occurring due to leptospirosis.

## Introduction

*Leptospira interrogans* (*L. interrogans*) are spirochetal bacteria responsible for a worldwide reemerging zoonosis [1]. Rodents asymptomatically carry the bacteria in their kidneys and excrete them in the urine, contaminating the environment. Humans become infected from contaminated ponds, through direct contact with the bacteria via broken skin or mucosa. Leptospirosis can be mild or severe with respiratory, liver and kidney failure, and constitutes a health problem is East Asia, especially among paddy workers. Chronic kidney disease (CKD) is a common feature of numerous renal diseases. A key component of CKD is renal fibrosis, a complex process involving different resident and infiltrated cell types and signaling pathways. Fibrosis results in structural and functional renal alterations characterized by an excessive accumulation of extracellular matrix proteins in scarring tissues, which may lead to organ dysfunction [1]. From human and canine biopsy studies, leptospirosis has been shown to be associated with chronic interstitial nephritis and fibrosis [2]. Recently, it was reported that leptospirosis led to irreversible tubule-interstitial fibrosis in a young male, requiring continuous hemodialysis [3]. In Taiwan, 10% of patients with CKD were seropositive for *L. interrogans*, although most of them did not have any history record of leptospirosis [4]. Therefore, underestimated leptospirosis could be one of the reasons for the high prevalence of kidney disease in Taiwan [4] and more generally in East Asia.

The physiopathology of leptospirosis has been studied in several animal models including rats, gerbils, guinea pigs, and hamsters, and revealed several decades ago that kidney tubulo-interstitial lesions were a hallmark of infection with *L. interrogans.* A recent study highlighted the importance of iNOS in tubulo-interstitial lesions in mice [5]. However, to our knowledge, the long-term pathophysiological consequences of *L. interrogans* infection in mice, and *in vivo* studies about *Leptospira*-induced fibrosis, have not yet been investigated. Nonetheless, *in vitro* studies showed that outer membrane components of *L. interrogans*, among them LipL32, the major lipoprotein of *L. interrogans* and TLR2 agonist [6], activate human cells [7] to produce extracellular matrix components [8], [9].

We recently developed a mouse model of acute leptospirosis. We showed that, in contrast to C57BL/6J mice that are asymptomatic, mice deficient for both Toll-like Receptor (TLR)-2 (TLR2) and -4 (TLR4) (TLR2/4dko) are susceptible, and can die from *L. interrogans* infection with all the features of the severe, acute human disease. We demonstrated that B cells, through both TLR2- and TLR4-mediated signaling, play a crucial role in clearance of the bacteria. Moreover, infected TLR2/4dko mice developed a deleterious inflammation within a few days, associated with renal tubulo-interstitial infiltrates of T cells [10]. We also recently showed that *L. interrogans* infection triggers a NLRP3-dependent IL1ß secretion in the mouse kidney, as a result of a synergistic effect of two cell wall components, leptospiral LPS and glycolipoprotein, through its downregulation of the Na/K ATPase pump [11]. Preliminary data obtained in surviving mice, several weeks after *L. interrogans* infection, suggested the presence of fibrotic lesions in mouse kidneys.

Innate immune receptors, TLRs and Nod-like receptors (NLRs) such as the inflammasome receptor NLRP3, have recently been shown to play a crucial role in the development and progression of tissue fibrosis of the lung [12], liver [13] and in a mouse model of kidney fibrosis induced by unilateral ureteral obstruction [14]. Whether innate receptors also play a role in murine *L. interrogans*-induced renal fibrosis and whether infiltrated inflammatory cells such as T cells or macrophages, already known to promote the renal fibrosis [15], [16], are important players in the *Leptospira* induced fibrosis is currently unknown.

Here, we characterized a novel murine model of renal fibrosis induced by bacterial infection, and showed that *Leptospira* infection of C57BL/6J mice led to a sustained fibrosis, associated with chronic carriage of *Leptospira*. Using several strains of transgenic mice, we determined that T cells and, unexpectedly, TLR and NLR receptors, were not required to generate *Leptospira*-induced fibrosis. However, we show that the iNOS enzyme, known to play a role in the interstitial nephritis due to *Leptospira*, also plays a role in the *Leptospira*-induced renal fibrosis.

## Materials and Methods

### Mice

Female C57BL/6J mice (8- to 10-wk old) were purchased from Janvier (Le Genest, France) and used as control mice. Mice deficient for TLR2 (TLR2ko), TLR3 (TLR3ko), TLR4 (TLR4ko), TLR5 (TLR5ko), TLR9 (TLR9ko) and MyD88 (MyD88ko), originally given by Shizuo Akira (Osaka University, Osaka, Japan), have been further backcrossed eight times into C57BL/6J mice and kindly provided by Michel Chignard (Institut Pasteur, Paris). Double TLR2/TLR4 deficient mice (TLR2/4ko) have been previously described [10]. Mice deficient for Nod1 (Nod1ko) or Nod2 (Nod2ko), respectively given by John Bertin (Millenium, Cambridge, MA) and Jean-Pierre Hugot (Hôpital Robert Debré, Paris, France) to Dana Philpott (University of Toronto, Toronto), have been further backcrossed eight times into C57BL/6J mice, before being crossed and genotyped to get double Nod1/Nod2 deficient mice (Nod1/2dko). CD3 deficient mice (CD3ko), B cell deficient mice (μMT), and caspase-1 deficient mice (Casp1ko) were kindly provided by Armelle Phalipon (Institut Pasteur, Paris), Claude Leclerc (Institut Pasteur, Paris), and Mathew Alberts (Institut Pasteur, Paris), respectively. iNOS deficient mice (iNOSko) in the C57BL/6J background were obtained from the Jackson Laboratory.

### Ethics statement

All protocols were reviewed by the Institut Pasteur, the competent authority, for compliance with the French and European regulations on Animal Welfare and with Public Health Service recommendations. This project has been reviewed and approved (# 2013-0034) by the Institut Pasteur ethic committee (CETEA #89).

### *L. interrogans* and in vivo infection experiments

*L. interrogans* serovar Copenhageni strain Fiocruz L1–130 and *L. interrogans* serovar Manilae strain L495 were used in this study as described earlier [11]. Just before infection, bacteria in early stationary phase (around 5×10^8^ *Leptospira* per ml), grown in liquid EMJH medium at 28°C, were centrifuged, resuspended in endotoxin-free PBS, and counted using a Petroff-Hauser chamber. The LD_50_ of the Fiocruz strain in C57BL6/J WT mice is above 10^9^ bacteria/mouse, and WT mice are considered resistant to *L. interrogans* infection, as are CD3ko, iNOSko, TLR3ko, Casp1ko and Nod1/2ko mice. However, the LD_50_ of the Fiocruz strain in sensitive MyD88ko, TLR4ko and TLR2/4ko mice is around 10^7^ bacteria/mouse. Therefore, to ensure survival of all mice, *Leptospira*-resistant mice were infected with 2×10^8^ Fiocruz strain in 200 µl of PBS by the intraperitoneal (IP) route, whereas *Leptospira*-sensitive mice were infected with a lower dose of 2×10^6^ Fiocruz/mouse. Since the LD_50_ of the Manilae strain in WT mice is around 10^8^ bacteria/mouse, WT mice were infected with 10^7^ Manilae strain/mouse. Mice were sacrificed at different days post-infection (p.i.). Liver, kidneys, and lungs of infected and naive mice were removed. Organs were either rapidly frozen in liquid nitrogen, then stored at −80°C for nucleic acid preparations, or fixed for histology and immunohistochemical studies.

### Antibiotic treatment

Within the first days after experimental IP infection, *Leptospira* disseminate through blood circulation and reach all the organs, including the kidneys. Then, within one week post-infection, the bacteria disappear from the circulation and settle in and colonize their renal niche, then begin to be shed in the urine. Therefore, penicillin G (Sigma), at the equivalent human dose of 9 million units/60 kg, was administered in 100 µl of endotoxin free PBS (Biowhittaker) to 20 g C57BL/6J mice via IP route once a day for 5 consecutive days, either beginning one day p.i. to clear disseminating bacteria, or 3 days p.i. to stop the infection after the renal colonization has started.

### Generation of mouse bone marrow-derived macrophages and in vitro stimulation

Bone marrow derived macrophages (BMDM) were isolated as described previously and cultured for 7 days in 10% L929-conditioned medium [11]. Mouse BMDM (2×10^5^ cells in 200 µl) were seeded in 96-well plate and stimulated 3 h later with live or killed (56°C 30 min) *L. interrogans*, at different multiplicities of infection (MOI). Stimulations were stopped 24 h later, and nitric oxide (NO) formation was evaluated in supernatants by the measure of nitrites (NO^2−^) via the Griess reaction.

### Plasma biochemical analysis

Before sacrifice, blood samples (200 µl) were collected by retromandibular puncture into tubes containing 20 µl of heparin (Choay). Samples were centrifuged (1500 g, 5 min), and the plasma was stored at −80°C. Total serum creatinine was measured in plasma samples using an Olympus AU400 autoanalyzer.

### Leptospiral load in urine

The leptospiral burden in urine was determined by quantitative real-time DNA PCR (qPCR). Total DNA was extracted from a drop of urine (5 to 100 µl) using the Maxwell 16 instrument and Cell LEV DNA purification kit (Promega). The qPCR reaction was calibrated using a known number of heat-killed *L. interrogans*. The DNA concentration was adjusted to around 100 ng in the qPCR reaction. Primers were designed in the peculiar *lpxA* gene of *L. interrogans* Fiocruz strain [17] to specifically detect pathogenic *Leptospira spp* but not other spirochetes or bacteria, using Primer Express 3 software (Forward (Fw): 5′-TTTTGCGTTTATTTCGGGACTT-3′; Reverse primer (Rv): 5′-CAACCATTGAGTAATCTCCGACAA-3′; Probe: 5′-TGCTGTACATCAGTTTTG -3′). qPCR reactions were run on a Step one Plus real-time PCR apparatus using the absolute quantification program (Applied Biosystems), with the following conditions: 50°C for 2 min, 95°C for 10 min, followed by 40 cycles with denaturation at 95°C for 15 s and annealing temperature 60°C for 1 min, according to the manufacturer's instructions. Results were expressed as the number of *Leptospira* in 100 µl of urine. Observation and subcultures of *Leptospira* from fresh urines showed that shed bacteria were mobile and alive (data not shown).

### Morphological and immunohistochemical studies

Thin transversal sections of kidneys were collected and fixed in Dubosq-Brazil for 2 h then post-fixed in 10% formalin in PBS and embedded in paraffin. Tissue sections (5 µm thickness) were stained with Hematoxylin-Eosin, to evaluate inflammatory changes by light microscopy, or labeled for 30 min with a solution of 0.1% (W/V) Red Sirius in saturated picric acid, for evaluation of the fibrosis. Picro-Sirius is currently used to stain collagens I and III deposited within the interstitial areas, and is recommended for the diagnosis of chronic renal injury. All sections were examined by two pathologists blinded to the experimental conditions. The degree of interstitial inflammation was graded on a 6-point scale as follows: 0- no inflammation, 1- scattered interstitial mononuclear inflammatory cells, 2- mild diffuse mononuclear cell infiltration, 3- focal nodular mononuclear cell infiltration, 4- diffuse and nodular mononuclear cell infiltration without tubulitis 5- diffuse and nodular mononuclear cell infiltration with significant tubulitis. The degree of interstitial fibrosis was determined using a semi-quantitative scale as previously established [18] as follows: 0- no abnormality, 1- slight increase of interstitial fibrosis affecting less than 25% of kidney samples, with almost normal tubule, 2- moderate interstitial fibrosis affecting less than 25–50% of kidney samples with focal tubular atrophy, 3- severe interstitial fibrosis affecting more than 50% of kidney samples with diffuse extensive tubular atrophy. Morphometry was performed using computerized automatic scan (Visilog, VFG, Paris) and expressed as the mean of Red Sirius positive labeling per surface area (10^4^ µm^2^), counted on five different kidney tissue sections for each condition tested.

Immunohistochemical studies were performed using avidin-biotin coupled to peroxidase substrate kits (Vector Laboratories) according to the manufacturer's instructions. Peroxidase activity was revealed with diaminobenzidine (brown staining) (Dako REAL detection System). Antibodies used in this study were: a polyclonal antibody against LipL32 (a kind gift from David Haake, 1/2000), a monoclonal antibody against CD3 (Santa Cruz Sc-20047, 1/100), a rat monoclonal anti-mouse-Gr1 (Ly-6G/C) antibody (CliniSciences 1/100) and a monoclonal antibody, anti CD11b (Clinisciences, 1/100). The number of labeled cells (T cells with anti CD3 antibody, neutrophils with anti Gr1 antibody, and macrophages/monocytes with anti CD11b) per surface area (10^4^ µm^2^) was counted on five different kidney tissue sections for each of the experimental condition tested.

### Transmission electronmicroscopy

Kidney samples were fixed for 30 min in 2.5% glutaraldehyde in 0.1 M cacodylate buffer, embedded in Epon, and processed for transmission electronmicroscopy by standard procedures.

### Real-time and reverse transcription PCR (qRT-PCR)

Total RNA was extracted from kidneys using the RNeasy mini kit (Qiagen). RNA concentration was determined by measuring optical density at 260 nm. 2 µg total RNA was incubated in a final volume of 20 µl containing 1 µl oligo dT (100 µM) (Fermentas), 1 µl dNTP (10 mM each), 2 µl DTT (0.1 M), 0.5 µl Superscript II reverse transcriptase (200 U/ µl) and 4 µl 5×first strand buffer (Invitrogen). RNA with oligo dT was first denatured at 65°C for 5 min, then the enzyme and other reagents were added and maintained at 42°C for 1 h, followed by heat-inactivation at 70°C for 15 min. The generated cDNA was stored at −20°C. After RT, qPCR was performed using cDNA combined with primers, probes and mixed according to the manufacturer's recommendations (Applied Biosystems). qPCR reactions were run on a Step one Plus real-time PCR apparatus (Applied Biosystems), with the following conditions : 50°C for 2 min, 95°C for 10 min, followed by 40 cycles with denaturation at 95°C for 15 s, and annealing temperature 60°C for 1 min. Data were analyzed according to the method of relative gene expression using the comparative *C*_T_ method also referred to as the 2^−ΔΔ*C*T^ method. PCR data were reported as the relative increase in mRNA transcripts versus that found in kidneys from naive mice, corrected by the respective levels of hypoxanthine-guanine phosphoribosyltransferase (HPRT) mRNA used as an internal standard. The sequences of primers and probes for iNOS, IL6, TNF, and RANTES have already been described [10]. Primers for TGFß (NM_011577) were Fw:5′-TGACGTCACTGGAGTTGTACGG-3′ (nt1461–1482), Rv: 5′-GGTTCATGTCATGGATGGTGC-3′ (nt 1610–1630), probe 5′-TTCAGCGCTCACTGCTCTTGTGACAG-3′ (nt 1522–1547). Validated primers and probes for Mmp2, ACTA-2 and fibronectin (Mn_00439498, Mn_01546133, Mn_01256744, respectively) were from Applied Biosystems.

### Rescue of μMT mice with serum

μMT mice lacking B cells were infected IP with 2×10^7^ *L. interrogans* Fiocruz strain and rescued from death by passive transfer of protective serum, as described [10]. To obtain the protective serum, ten C57BL/6J mice were infected with 5×10^7^ *L. interrogans* serovar Fiocruz and bled 20 days p.i. After overnight coagulation at 4°C, the sera were collected and heat-inactivated at 56°C for 30 min and kept frozen at −80°C. To be sure the *Leptospira* will reach the kidney, we let the infection develop for two days before injecting IP 200 µl of protective pooled sera to both infected and naive μMT mice. Survival was monitored and all surviving mice were sacrificed at 15-days p.i.

### Statistical analysis

Statistical analysis was performed using GraphPad Prism software. The unpaired *t* test, (two-tailed P values) was used to compare two groups. The distribution of three or more groups was analyzed by One-Way ANOVA. Values are expressed as means, or means + standard deviation (SD). A *p* value<0.05 was considered significant.

## Results

### *Leptospira* infection triggers a sustained fibrosis in the mouse kidney

**Figure 1:**
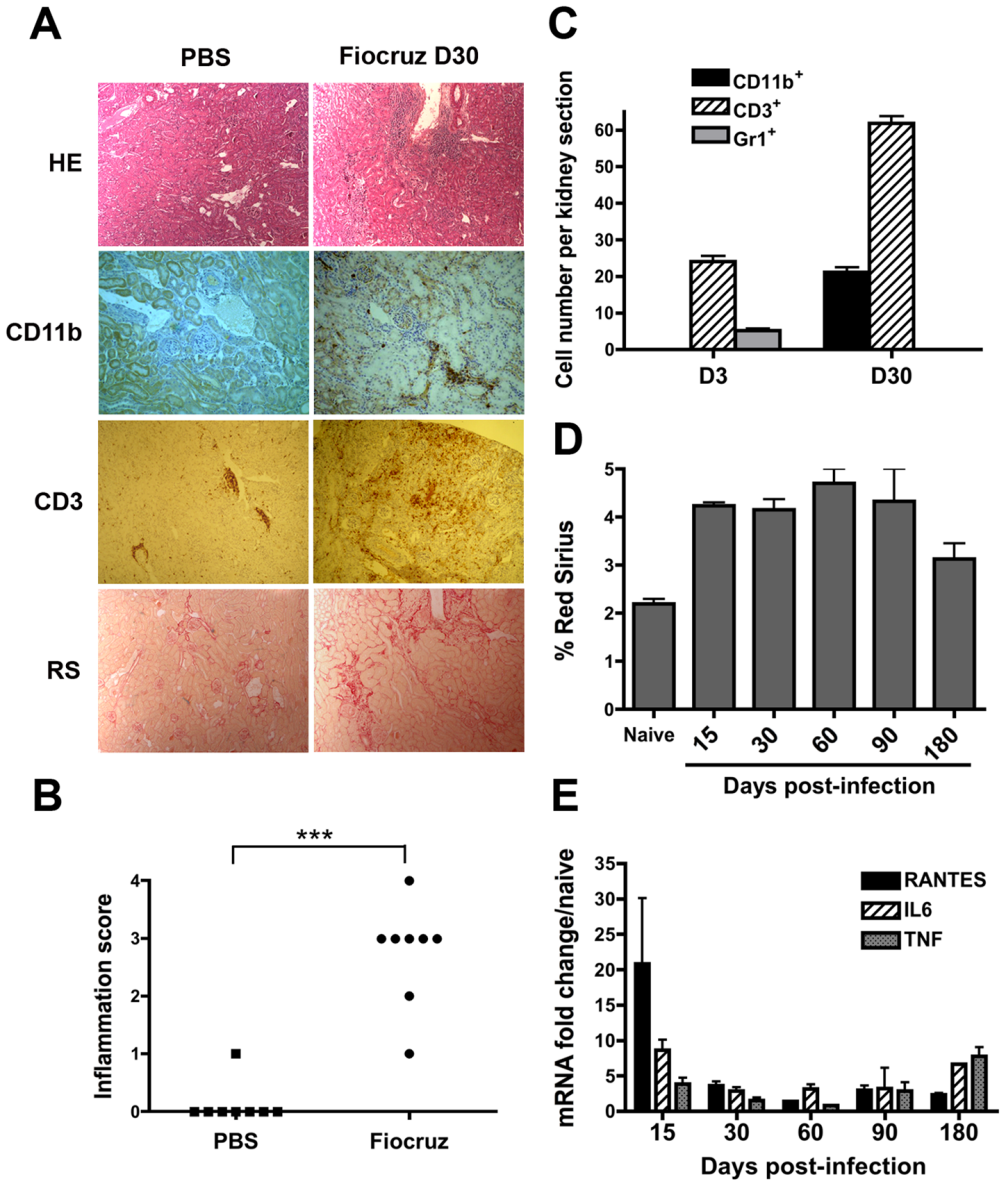
*Leptospira* infection triggers inflammation and fibrosis in the mouse kidney. (A) Light microscopy of nodular infiltrates stained with hematoxylin-eosin (HE), infiltrating CD11b^+^ macrophages and CD3^+^ T cells and collagen deposition stained with Red Sirius (RS) in kidneys from C57BL/6J mice 30 days (D30) after the inoculation of 2×10^8^ *L. interrogans* strain Fiocruz. As controls, mice were injected with PBS. Magnification, ×100. (B) Score of kidney inflammation of interstitial nodular infiltrates per surface areas from five different renal tissue sections in control (PBS) and *L. interrogans* strain Fiocruz infected mice (n = 8 per group). (C) Quantification of the number of CD11b^+^ macrophages, Gr1^+^ neutrophils and CD3^+^ T Lymphocytes per surface area in kidneys from day-3 (D3) and day-30 post-infected mice. (D) Fibrosis quantification by Red Sirius morphometry, expressed as percent of surface area and (E) Inflammation evaluation by mRNA expression of proinflammatory mediators in kidneys of 10 infected mice sacrificed at different time points. Values are means ± SD of counts (C and D) and mRNA quantification (E) from 5 different tissue sections from n = 2 separate mice in each group tested. ***P<0.001.

C57BL/6J mice infected with 2×10^8^ *L. interrogans* serovar Copenhageni strain Fiocruz are resistant, and survive infection [10]. One month p.i., WT mice were sacrificed and their liver, lungs, and kidneys studied by immunohistochemistry. Neither inflammation nor fibrosis could be observed in the livers or lungs of infected mice (data not shown), although infected mice displayed kidney inflammation and fibrosis (Fig. 1A). Indeed, hematoxylin-eosin staining revealed from 1 to 4 inflammatory foci, composed of around one hundred infiltrating cells per kidney section in infected mice, compared to naive mice injected with PBS, that showed no inflammation (Fig. 1A and Fig. 1B). The cellular composition of the infiltrates in naïve and infected mice was investigated by immunohistochemistry. One month p.i., the renal infiltrates were mostly composed of CD3 positive (^+^) T cells and CD11b^+^ macrophages/monocytes. In contrast, the few Gr1^+^ cells (mostly neutrophils) detected at day-3 p.i. were no longer detected in kidneys one month p.i. (Fig. 1C). Red Sirius labels collagens I and III, known to accumulate upon fibrosis. The observation and morphometry of Red Sirius staining one month p.i., revealed mild focal fibrosis in both the cortex and medulla regions of the kidney in *Leptospira-*infected mice (Fig. 1A and Fig. 1D). To ensure that fibrosis was not peculiar to the Fiocruz strain, C57BL/6J mice were infected with another pathogenic *L. interrogans*, serovar Manilae strain L495. One month p.i., similar mild fibrosis was observed in kidneys from mice infected with the serovar Manilae strain L495 (data not shown).

We next thought to establish the kinetics of fibrosis appearance and potential healing in mice. C57BL/6J mice were infected with *L. interrogans* copenhageni strain Fiocruz and sacrificed at 15, 30, 60, 90, and 180 days p.i, and their kidneys analyzed by Red Sirius morphometry and qRT-PCR to detect fibrosis and inflammation, respectively. The fibrosis was already present at day-15 p.i., and persisted at the same mild level for the next 3 months (Fig. 1D). Fibrosis was still present after 6 months p.i., although a tendency to decrease was observed. Because fibrosis usually occurs upon inflammation, pro-inflammatory chemokines (RANTES) and cytokines (IL6, TNF) were monitored by qRT-PCR. The leptospiral infection triggered a marked inflammatory response in day-15 p.i kidneys that decreased over time, but unexpectedly reappeared at day-180 p.i. (Fig. 1E). We also measured the expression of TGF-ß that is usually upregulated upon fibrosis, but we did not find any significant upregulation in the kidneys of infected mice (data not shown). These findings suggest that *Leptospira* infection triggers an early inflammation, associated with a sustained renal fibrosis for at least 3 months p.i.

### Early antibiotic treatment abolishes the *Leptospira*-induced fibrosis

**Figure 2:**
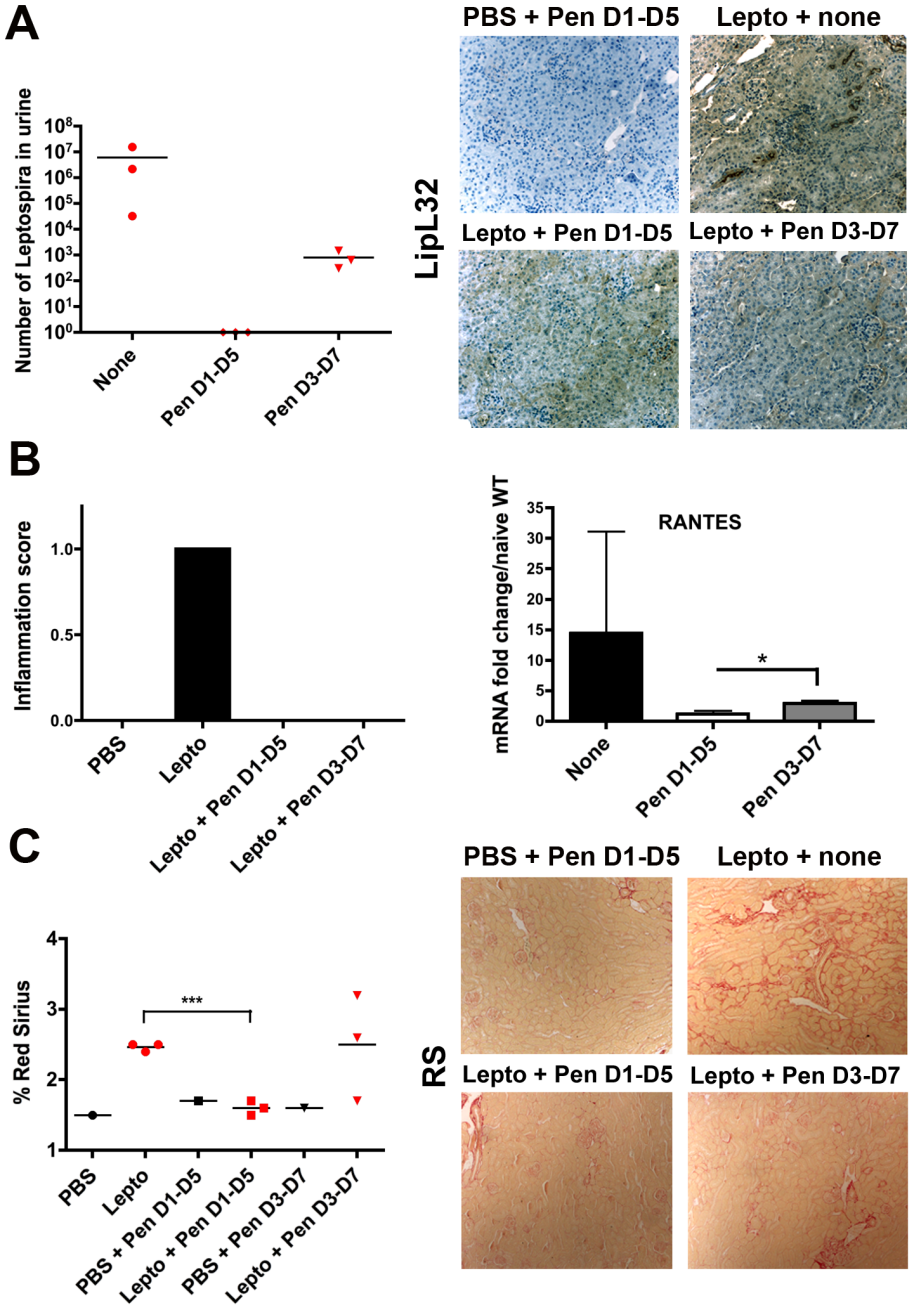
Effects of antibiotic treatment on *Leptospira*-infected mice. C57BL/6J mice were infected with 10^7^ *L. interrogans* serovar Manilae (n = 3) or PBS (n = 1) and injected (IP) daily for 5 days with penicillin G (Pen) from day-1 p.i. until day-5 (D1–D5) or from day-3 p.i. until day-7 (D3–D7). Thereafter, mice were sacrificed at day-24 p.i. (A) Leptospiral loads in 100 µl urine determined by qRT-PCR, and imaging of the LipL32 leptospiral major antigen immunostaining in kidney sections. (B) Inflammation score (left panel) and pro-inflammatory RANTES mRNA expression (right panel). (C) Quantification (left panel) and microscopy (right panels) of fibrosis by Red Sirius staining. Values are counts (The bars represent the mean value in each group) or means of mRNA quantification ± SD from n = 3 mice per group and are from one representative experiment of two. *P<0.05; ***P<0.001 between groups.

Since fibrosis is a complex mechanism observed in many inflammatory conditions, most of them unrelated to infectious processes, we wondered whether the presence of *Leptospira* or their antigens in the kidney were required for inducing fibrosis. Therefore, C57BL/6J mice were infected with *L. interrogans* serovar Manilae and then treated daily for five consecutive days with penicillin G, the antibiotic used to treat most patients with leptospirosis. Infected mice were either treated from day-1 to day-5 p.i. (D1–D5), allowing sufficient time for the bacteria to disseminate and reach the kidneys, but be cleared before colonization, or treated from day-3 until day-7 (D3–D7) to allow some *Leptospira* to colonize the kidneys before the antibiotic treatment. Thereafter mice were sacrificed at 24-day p.i., and renal fibrosis and inflammation quantified by Red Sirius morphometry and scoring (data not shown). In parallel, the carriage of *Leptospira* was detected by qPCR of leptospiral DNA in the urine. High amounts of *Leptospira* were detected in the urine of non penicillin-treated infected mice, and to a much lesser extent in the urine of D3–D7 antibiotic-treated mice, whereas no *Leptospira* were detected in the urine of D1–D5 antibiotic-treated mice (Fig. 2A, left panel). In parallel, the presence in the kidneys of leptospiral antigens was monitored by immunohistochemistry using an antibody directed against LipL32, the major lipoprotein of *Leptospira* (Fig. 2A, right panel). Interestingly, LipL32 was detected in all infected mice, with a more pronounced labeling in the infected, non-antibiotic treated mice, confirming that the timing of penicillin treatment was adequate to allow *Leptospira* to reach the kidneys. Histological scoring also revealed that the inflammation was only present in infected, non-treated mice and was not observed in D1–D5 or D3–D7 antibiotic-treated mice (Fig. 2B, left panel). Also, qRT-PCR analysis revealed less up-regulation of the inflammatory RANTES mRNA in antibiotic-treated mice compared to the infected mice, not treated with antibiotics. D3–D7 penicillin-treated mouse kidneys exhibited significantly greater levels of RANTES mRNA expression than D1–D5 penicillin-treated mouse kidneys (Fig. 2B, right panel). This suggests that the inflammation observed in the kidney is related to the *Leptospira* load in the urine, reflecting the renal burden (data not shown). Red Sirius staining (Fig. 2C, right panel) and quantification (Fig. 2C, left panel) revealed a slight renal fibrosis in non-treated mice compared to naïve mice, although no fibrosis was observed in D1–D5 antibiotic-treated mice, and 2 out of 3 mice exhibited fibrosis in the group of D3–D7 penicillin-treated mice. These results suggest that renal fibrosis occurs in mice colonized by *Leptospira* in their kidneys and excreting live *Leptospira*. However, no correlation between the number of *Leptospira* in the urine and the extent of fibrosis could be observed. Of note, no inflammation or renal fibrosis were observed in the kidneys of mice that cleared the *Leptospira* infection, but still harbored leptospiral antigens.

### T and B lymphocytes cells are not required for *Leptospira*-induced renal fibrosis

**Figure 3:**
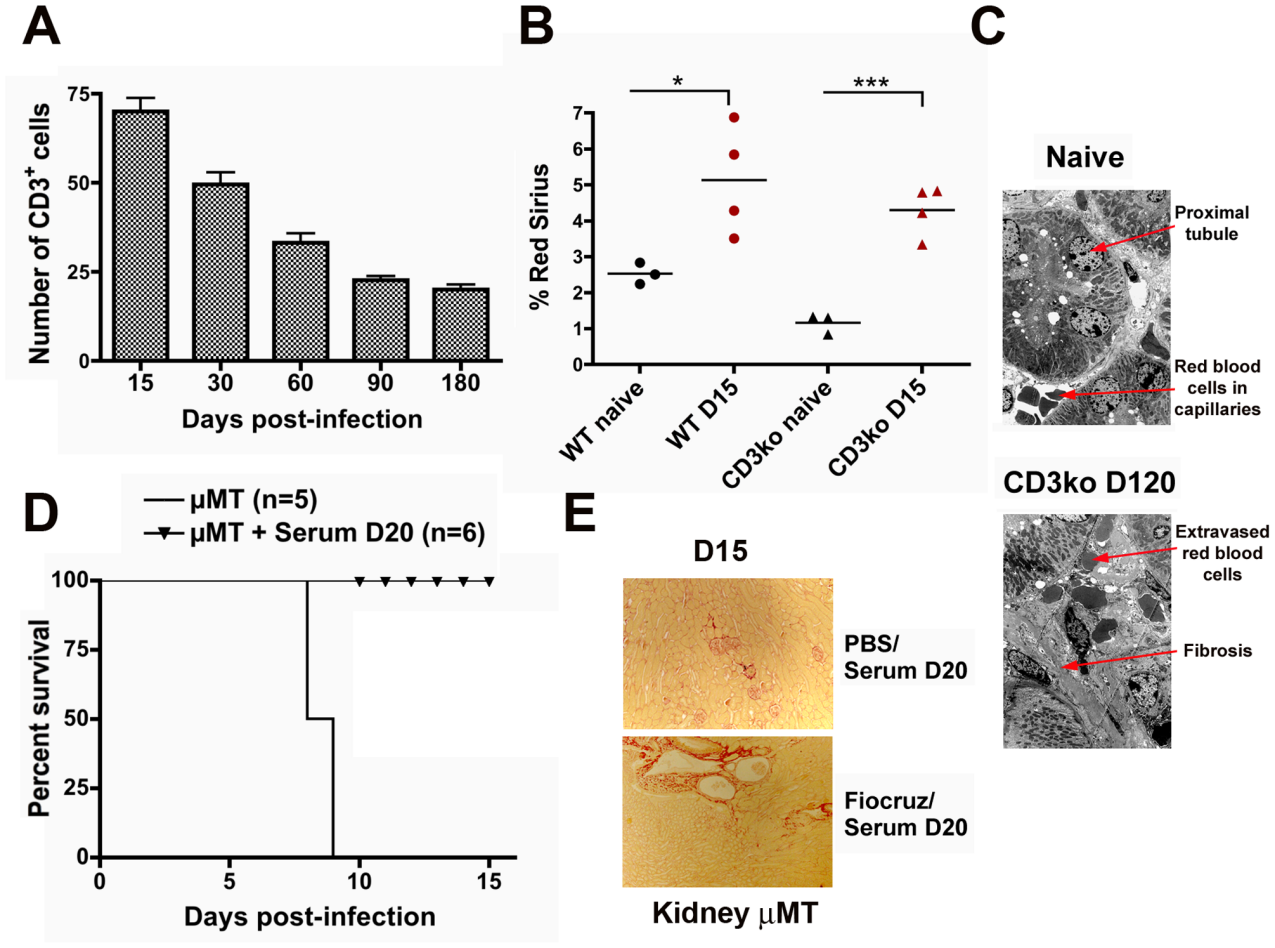
T and B cells are not involved in *Leptospira*-induced renal fibrosis. (A) Quantification of the number of infiltrating CD3^+^ cells in *Leptospira*-infected kidneys from WT C57BL/6J mice at different time points post p.i.. Values are means ± SD from five kidney sections per surface area from (n = 2) mice at each different time point tested. (B) Percentage of Red Sirius labeling per surface area in kidney sections from WT and CD3ko mice at day-15 p.i. (D15) and in naive mice. The bars represent the mean value in each group. (C) Electron microscopy (magnification ×100) of kidneys sections from a naïve mouse and CD3ko mouse at 4 months p.i. (D120) showing fibrosis. (D) Survival curves and (E) images of renal Red Sirius staining from B cell deficient naïve μMT mice or infected with 10^7^ *L. interrogans* strain Fiocruz, rescued by immune serum from *Leptospira*-infected WT mice at day-15 p.i. (D15). *P<0.05; ***P<0.001 between groups.

We next thought to assess the role of the inflammation in *Leptospira*-induced renal fibrosis. Because inflammatory infiltrates observed one month p.i. with *L. interrogans* in kidneys were mostly composed of T cells (see Fig. 1C), we questioned whether T cells could be involved in the *Leptospira*-induced fibrosis. Indeed, T cells have been recently shown to promote renal fibrosis induced by unilateral ureteral obstruction in mice [15]. First, semi-quantitative evaluation by immunohistochemistry of the number of T cells in kidneys of infected C57BL/6J mice revealed that the number of T cell infiltrates decreased over time (Fig. 3A). This suggests that T cells are not directly associated with the observed sustained renal fibrosis. However, to be sure that T cells infiltrates were not the initial trigger of fibrosis, mice deficient for T cells (CD3ko mice) and their C57BL/6J counterparts were infected with *L. interrogans* strain Fiocruz. Mice were sacrificed at day-15 p.i. and their kidneys prepared for immunohistochemistry. Red Sirius morphometric quantification was equivalent in infected WT and CD3ko mouse kidneys, which were both significantly greater compared to the respective naive kidneys (Fig. 3B). Electron microscopy analysis of the kidney of a 4 month post-infected CD3ko mouse revealed a marked interstitial fibrosis, in contrast to the morphological aspect of the kidney of a naive mouse (Fig. 3C). Altogether, these results strongly suggest that T cells do not participate in the induction of renal fibrosis caused by *Leptospira*.

B cells from the adaptive immune response have also been reported to be involved in renal fibrosis through IgG deposition [19]. We already showed that B cells were crucial for clearance of *Leptospira*, and that transgenic mice deficient for B cells (μMT mice) were lethally susceptible to experimental leptospirosis [10]. To test the role of B cells in *Leptospira*-induced renal fibrosis, μMT mice were infected with *Leptospira* and rescued with passive transfer of protective serum obtained from *Leptospira*-infected C57BL/6J mice 20 days p.i. (Fig. 3D), as previously described [10]. Serum-treated naïve and infected μMT mice were sacrificed at day-15 p.i. Kidneys of rescued mice, stained with Red Sirius, showed some mild fibrosis compared to kidneys from the non-infected mice treated with protective serum (Fig. 3E), suggesting that B cells are not involved in *Leptospira*-induced fibrosis. Collectively, these results suggest that the adaptive immune response to *Leptospira* from both T and B cells is not directly involved in the induction of renal fibrosis.

### *Leptospira*-induced renal fibrosis does not depend on TLR2 and TLR4

**Figure 4:**
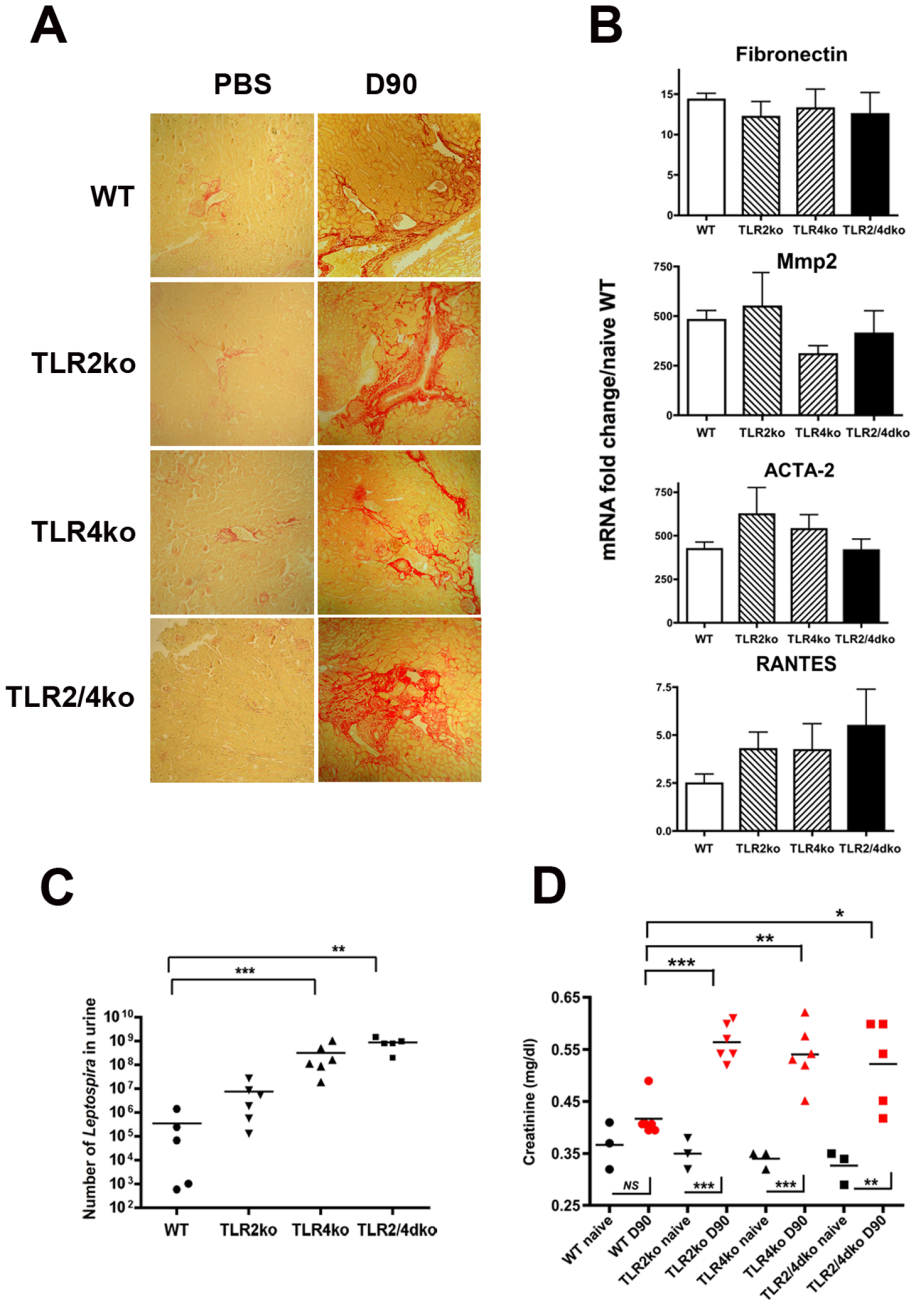
TLR2 and TLR4 are not involved in *Leptospira*-induced fibrosis. (A–D) Renal fibrosis, levels of inflammatory mediators, bacterial loads, and serum creatinine levels in WT or TLR2 and/or TLR4 deficient mice infected with 2×10^6^ *L. interrogans* Fiocruz at 3 months p.i. (D90) (A) Red Sirius staining. (B) Fibrosis and inflammation evaluation by mRNA expression of fibronectin, Mmp2, ACTA-2 and RANTES, in kidneys of WT, TLR2ko, TLR4ko and TLR2/4dko mice at 3 months p.i. Values are means ± SD from n = 5 mice per genotype group. No statistical difference between genotypes was found for the different markers by One-Way Anova. (C) Bacterial loads in 100 µl of urine from mice at 3 months p.i. The bars represent the mean values in each group tested. (D) Serum creatinine levels in naïve and *Leptospira*-infected mice at 3 months p.i. *P<0.05; **P<0.01; ***P<0.001 between groups.

Innate immunity receptors have recently been linked to fibrosis. Since TLR2 and TLR4 are crucial in mouse defense against *Leptospira* [10], and since *in vitro* experiments suggest that TLR2 stimulation by outer membrane components of *Leptospira* is important for expression of fibronectin and extra cellular matrix components [9], we aimed at testing whether TLR2 and/or TLR4 could be involved in *Leptospira*-induced renal fibrosis in mice. We previously showed that TLR2/4dko mice are sensitive to pathogenic *Leptospira* and die from the infection. Therefore, groups of WT, TLR2ko, and TLR2/4dko mice were infected with a sub-lethal dose (2×10^6^) of *L. interrogans* strain Fiocruz. Mice were then sacrificed at day-90 p.i., and their kidneys analyzed by Red Sirius morphometry. Unexpectedly, *Leptospira*-infected kidneys from all genotypes, including TLR2/4dko mice, presented fibrosis when compared to their naïve counterparts (Fig. 4A). Consistent with the marked Red Sirius staining in kidneys from all tested mice, mRNA expression of typical markers classically up-regulated in fibrotic conditions, such as metalloprotease 2 (Mmp2) [20] which is important for the degradation of extracellular matrix components, smooth muscle actin (ACTA-2) over-expressed by activated myofibroblasts [16] and fibronectin, an extracellular matrix glycolipoprotein that binds other fibrillar components such as collagens, were all up-regulated in the kidneys from mice at day-90 p.i., with no significant statistical differences between the four 4 different mouse genotypes (Fig. 4B). Up-regulation of mRNA expression of the inflammatory RANTES chemokine was also detected in kidneys from all 4 mouse groups, with no statistical differences between groups (Fig. 4B). These rather unexpected findings, showing no differences between *Leptospira*-induced fibrosis and inflammation in WT *versus* TLR deficient mice, led us to measure the leptospiral loads in the urine of infected mice. Results from q-PCR of the leptospiral DNA showed that 90 days p.i. WT and TLR2ko mice excreted around 10^5^ and 10^7^ *Leptospira* per 100 µl of urine respectively, whereas susceptible TLR4ko and TLR2/4dko mice were more heavily infected, with around 10^8^ and 10^9^ *Leptospira* per 100 µl of urine respectively (Fig. 4C). Of note, quantification of renal burden in the corresponding kidneys by q-PCR of the leptospiral DNA gave the same trend (data not shown). These data are consistent with our previous results obtained at day-3 p.i., showing an important role of TLR2 and TLR4 in the mouse defense and clearance of *Leptospira* [10], and further suggest that the extent of fibrosis is not directly proportional to bacterial excretion, reflecting the renal bacterial loads. To evaluate whether *Leptospira* infection, and subsequent renal colonization and fibrosis could be deleterious to renal function, the level of serum creatinine, used as a marker of renal function, was measured in the serum of mice at day-90 p.i. Interestingly, the infected WT mice did not show any statistically significant elevation of the serum creatinine when compared to naïve WT mice (Fig. 4D). In contrast, all TLRko mice presented a slight but significant elevation of the serum creatinine levels compared to those of WT mice and corresponding naïve TLRko mice (Fig. 4D). These results indicate that neither TLR2 nor TLR4 were required for *Leptospira*-induced renal fibrosis, and suggest that the chronic carriage of *Leptospira* can be associated with a slight alteration of the kidney function, if the *Leptospira* load is not restricted by the presence of TLR2 and/or TLR4.

### TLR and NLR independent *Leptospira*-induced fibrosis

**Figure 5:**
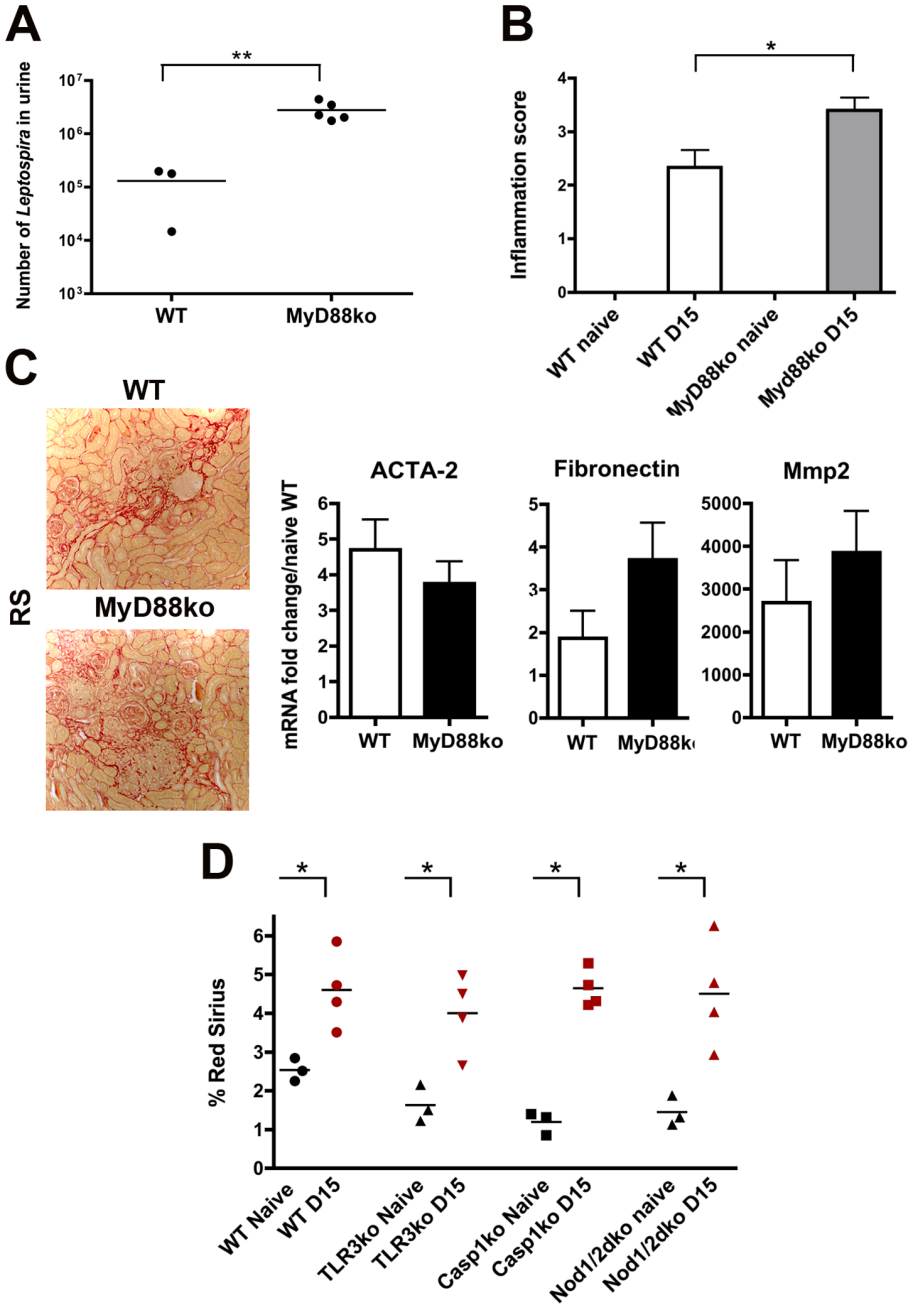
TLRs and NLRs are not involved in *Leptospira*-induced fibrosis. WT and Myd88ko mice infected with 2×10^6^ *L. interrogans* Fiocruz were sacrificed at 15 days p.i. (A) Bacterial loads in 100 µl of urine. (B) Inflammation score in kidneys. (C) Red Sirius staining (C, left panel) and mRNA expression of ACTA-2, fibronectin and Mmp2, in the infected WT and Myd88ko mice kidneys (C, right panel). Data are means ± SD from WT mice (n = 3) and MyD88ko mice (n = 5). The images in C are representative of three separate experiments. (D) Percentage of Red Sirius staining in kidneys from WT mice, TLR3ko, Casp1ko and double Nod1/2ko mice infected with 2×10^8^ *L. interrogans* Fiocruz and sacrificed at day-15 p.i. (n = 4 per genotype group). Three naïve kidneys of each genotype were used as controls. *P<0.05; **P<0.01 between groups.

Aside from TLR2 and TLR4, other TLRs that have not yet been studied in the context of leptospirosis, such as TLR5 that senses flagellin, and TLR9, the receptor of bacterial DNA, could in theory be involved in the murine defense against *L. interrogans*. MyD88 is the adaptor of most TLRs, except TLR3. To get insight in the putative role of TLRs other than TLR2 and TLR4 in the *Leptospira*-induced fibrosis, MyD88ko mice were infected with a sub-lethal dose (2×10^6^ bacteria) of *L. interrogans* strain Fiocruz. Fifteen days p.i., a greater burden of *Leptospira* was detected in the urine (Fig. 5A), and an increased renal inflammatory response measured in kidneys from MyD88ko mice compared to WT mice (Fig. 5B). Red Sirius staining also revealed a fibrosis in kidneys from both infected WT and MyD88ko mice (Fig. 5C, left panel), which was confirmed by qRT-PCR of different markers of fibrosis, whose up-regulation was not statistically different between WT and MyD88ko kidneys from infected mice (Fig. 5C, right panels). Infection of both TLR5ko and TLR9ko mice also confirmed that the *Leptospira*-induced fibrosis was independent of these TLRs (data not shown).

TLR3 is the only TLR using TRIF, but not MyD88, as an adaptor. TLR3 is known as a viral RNA sensor and is not expected to be involved in *Leptospira* defense. However, to be sure that none of the TLRs were involved in *Leptospira*-induced fibrosis, TLR3ko mice were infected with 2×10^8^ *L. interrogans* Fiocruz and sacrificed at day-15 p.i. Compared to naïve mice, *Leptospira* infection induced mild renal fibrosis in most of the infected TLR3ko (Fig. 5D). Collectively, these data indicate that none of the TLRs are critically involved in renal fibrosis caused by *Leptospira*.

Since TLRs seem not to be involved in *Leptospira*-induced renal fibrosis, we wondered whether other innate immune receptors from the NLR family, such as the cytosolic Nod1, Nod2, and NLR3 receptors, could be involved. Nod1 and Nod2 are receptors of muropeptides of bacterial peptidoglycan, but their role in defense against *Leptospira* remains unknown. We recently showed that *L. interrogans* activates the NLRP3 inflammasome in the mouse kidney [11]. When activated, the NLRP3 inflammasome induces caspase 1 cleavage, which in turns cleaves pro-IL1ß, allowing for its maturation and secretion. To test the role of these NLRs in the induction of renal fibrosis, WT, Nod1/2dko and Casp1ko mice were infected with 2×10^8^ *L. interrogans* strain Fiocruz (Fig. 5D). Compared to infected WT mice, no reduction in *Leptospira*-induced fibrosis was observed in kidneys of either Nod1/2dko or Casp1ko mice, showing that Nod1, Nod2 and NLRP3 are not involved in the *Leptospira*-induced renal fibrosis. As a whole, these results indicate that the fibrosis induced by *Leptospira* does not directly rely on TLR and NLR activation.

### iNOS participates in the *Leptospira*-induced fibrosis

**Figure 6:**
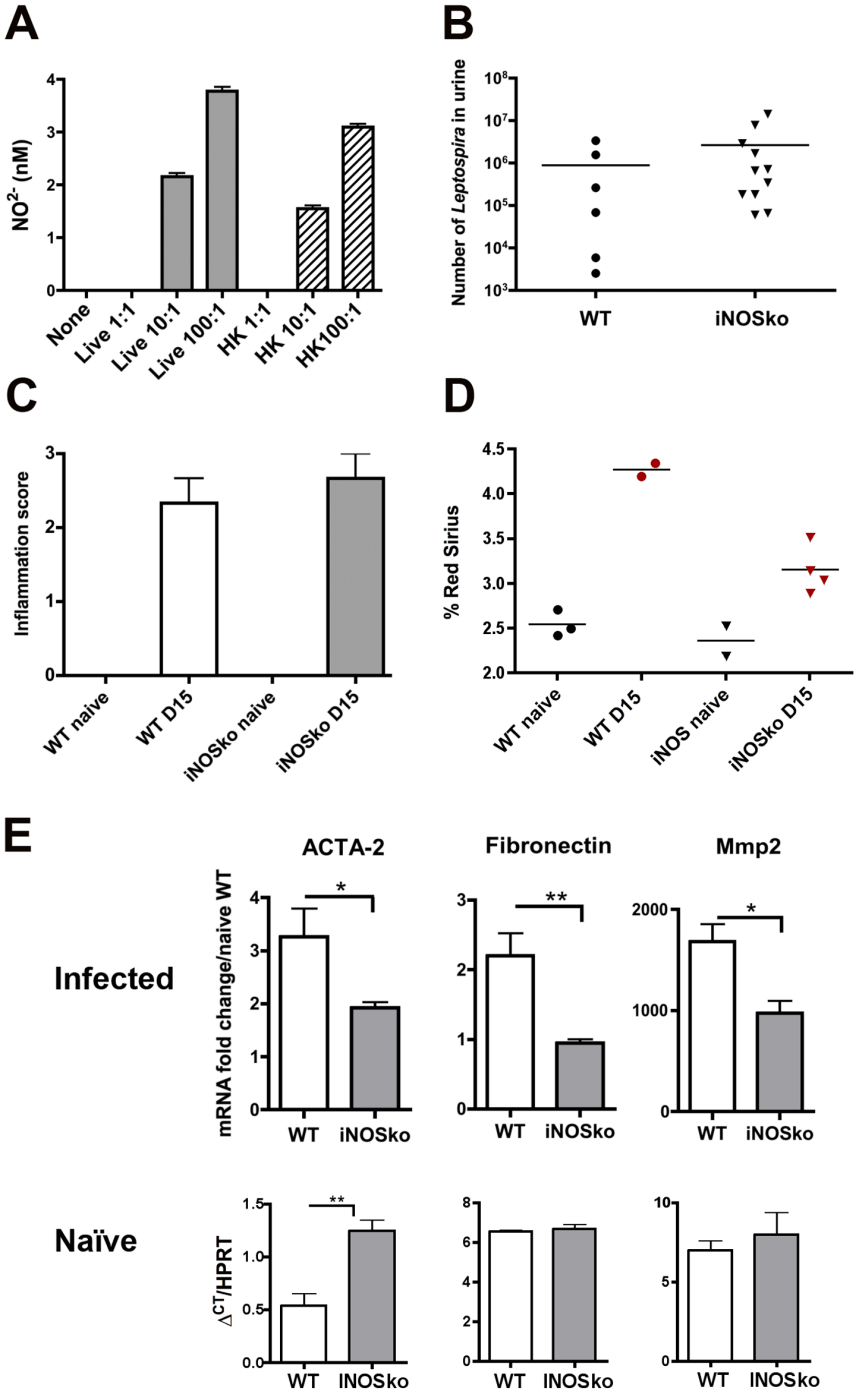
iNOS is involved in *Leptospira*-induced renal fibrosis. NO2^−^ production in supernatants from bone marrow macrophages derived from C57BL/6J mice, stimulated for 24 h with live (Lepto) or heat-killed (HKLepto) *L. interrogans* strain Fiocruz at a multiplicity of infection of 1 (1∶1) 10 (10∶1) and 100 (100∶1), as measured by the Griess reaction. Values are means +SD from 3 separate experiments. (B–E) Bacterial loads in urine (B), renal inflammatory scores (C), percentage of Red Sirius staining (D) and mRNA expression of ACTA-2, fibronectin and Mmp2 (E, upper panel) in kidneys from WT and iNOSko mice infected with 2×10^8^ *L. interrogans* Fiocruz and sacrificed at day-15 p.i.. Values are means ± SD from WT (n = 3) and iNOSko (n = 5) mice. (E, lower panel), comparison of mRNA expression levels of fibrosis markers in kidneys of naïve WT and iNOSko mice, measured as Δ^CT^ compared to HPRT. Values are means ± SD from WT (n = 3) and iNOSko (n = 3) mice. *P<0.05; **P<0.01 between groups.

Infiltrating CD11b^+^ macrophages were detected in kidneys of WT mice one month p.i. (see Fig. 1C). Their role in leptospirosis is difficult to assess since transgenic mice devoid of macrophages were not available. Apart from their phagocytic role, another defense mechanism of macrophages is the production of reactive oxygen species derived from nitric oxide (NO), which is toxic for bacteria and is produced by different nitric oxide synthase enzymes, among them the inducible iNOS. We previously showed that infection with *L. interrogans* strain Fiocruz induces iNOS mRNA upregulation in WT mouse kidneys at day-3 p.i. [10]. Here, BMDM from C57BL/6J mice were stimulated with *L. interrogans* strain Fiocruz, and nitrite (NO^2−^) production measured in cell supernatants 24 h later. Both live bacteria and heat-killed *Leptospira* induced dose-dependent production of NO^2−^ (Fig. 6A), showing that macrophages could be a potential source of NO induced by *Leptospira* infection. NO production constitutes an innate defense mechanism, but is also known to be responsible for cell toxicity. Recently iNOS was shown to play a deleterious role in *Leptospira*-induced interstitial nephritis [5]. WT and iNOSko mice were therefore infected with 2×10^8^ *L. interrogans* Fiocruz strain to test the role of iNOS in the induced fibrosis. Mice were sacrificed at day-15 p.i. and kidneys were processed for immunohistochemistry. No difference could be observed in bacterial loads in urine or inflammatory scores in kidneys between the infected WT and iNOSko mice (Fig. 6B and 6C), although renal fibrosis was slightly reduced, but not abolished, in the infected iNOSko compared to WT kidneys (Fig. 6D). Since the fibrosis at day-15 p.i. was mild, we also compared by qRT-PCR the expression of ACTA-2, fibronectin and Mmp2. These markers were statistically slightly less up-regulated in the kidneys of infected iNOSko mice compared to those of WT mice (Fig. 6E, upper panel), although expression levels of transcripts in kidneys of naïve iNOSko mice can not account for the observed down-regulation (Fig. 6E, lower panel). Altogether, these results suggest that upregulation of iNOS mRNA in response to *Leptospira* infection is deleterious and participates in induction of renal fibrosis. Nevertheless, additional mechanisms appear to be required for *Leptospira*-induced renal fibrosis.

## Discussion

Wistar rats are currently used as a model of chronic leptospirosis. After being infected, rats carry *Leptospira* in their kidneys, and persistently excrete them in their urine [21], [22]. Histopathology studies have revealed the presence of renal tubulo-interstitial lesions in all experimental animal models of acute and chronic leptospirosis [23]. Naturally susceptible C3H/HeJ mice develop acute renal nephritis after experimental infection with *Leptospira*, but, to our knowledge, chronic carriage of *Leptospira* for several months in mice has not been described [24]. Despite a few previous studies mentioning renal fibrosis in dogs [2] and rats [23] infected with *Leptospira*, the putative mechanisms leading to renal fibrosis induced by chronic carriage of *Leptospira* have not been investigated. In the present study, we characterized a mild, but sustained, renal interstitial fibrosis occurring upon experimental infection of C57BL/6J mice with *L. interrogans*.

A number of immunohistochemical studies have revealed the presence of *Leptospira* antigens including lipopolysaccharide (LPS), glycolipoprotein and lipoproteins in the renal tubulo-interstitial lesions, although the presence of live *Leptospira* was difficult to assess because of their long generation time and fastidious *in vitro* growth. Recent availability of quantitative real-time PCR techniques to evaluate the DNA presence in urine and organs eased the monitoring of *Leptospira* carriage. Visual and morphometric analysis of the kidneys of infected mice also permitted analysis of the degree of renal inflammation and fibrosis as described earlier [25]. Because fibrosis was found to be highly focal by visual examination, morphometric analysis revealed only an average mild fibrosis. However, this mild fibrosis was reproducibly measured and correlated with the mRNA upregulation of key markers of renal fibrosis. Although *Leptospira*-induced renal fibrosis appears to be correlated with inflammation as assessed by semi-quantitative scoring and upregulation of inflammatory cytokines, we did not find any significant up-regulation of TGF-ß (not shown), which is considered a key pro-fibrotic factor, usually produced by CD11b^+^ macrophages infiltrating the infected kidneys. In this line, a recent work showed that TGF-ß, mostly produced by infiltrating macrophages was not mandatory to ischemia/reperfusion induced fibrosis [26].

Acute leptospirosis is characterized by multiple organ failure, including liver, lung, and kidney dysfunctions, and marked inflammation and dissemination of *Leptospira* in all these organs [10], [24], [27], [28]. Interestingly, fibrosis was not found in the liver and lungs of infected mice that, contrary to the kidneys, were devoid of bacteria (not shown). This suggests that it is neither the initial phase of hematogenous dissemination of *Leptospira*, nor the initial inflammation, but the leptospiral colonization of the kidneys that triggers the fibrosis. In agreement with this hypothesis, mice that received the early antibiotic treatment (day-1 to -5) were cleared of *Leptospira* and did not develop renal fibrosis. In contrast, when the antibiotic treatment began later at day-3 p.i., it did not succeed in totally eliminating the *Leptospira*, perhaps due to their potential intracellular location in renal epithelial cells or protected niche in the lumen of renal tubules, and some of the mice developed renal fibrosis. These findings suggest that a direct correlation may exist between renal fibrosis and the presence of live *Leptospira* in the urine.

An unexpected and puzzling finding of this study was the lack of correlation between the levels of colonization and the extent of renal fibrosis. Indeed, the sensitive TLR4ko and TLR2/4dko mice, more heavily infected than the WT and TLR2ko mice, did not show any enhanced fibrosis. Together with our results on the antibiotic treated mice, the number of bacteria colonizing the kidney did not correlate with the extent of fibrosis. Therefore, we hypothesize that the initial endothelial insult of live *Leptospira* penetrating in the kidney may trigger the fibrosis, and that once within its niche in the kidney, colonization by *Leptospira* would not affect the fibrosis course.

Interestingly, one month p.i., the early antibiotic-treated mice developed neither renal fibrosis nor inflammation, although they harbored LipL32 antigens in the kidneys. LipL32 is the major lipoprotein of *Leptospira* and was demonstrated to be a TLR2 agonist [6], and an important component of the outer membrane, involved *in vitro* in the production of extracellular matrix components by human renal cell lines [8], [29]–[31]. Our present finding, together with the fact that TLR2 is not involved in *Leptospira* induced renal fibrosis, strongly suggests that *in vivo*, LipL32 is not involved in the fibrogenesis process. This discrepancy between the *in vivo* and in *vitro* results is striking and may emphasize that the complex phenomenon of fibrogenesis cannot be fully mimicked *in vitro*, and/or that species specificities of the TLRs may be involved. However, if differences in human and mouse TLR4 specificity towards leptospiral lipid A have already been shown [32], with only mouse TLR4 recognizing the lipid A, we never noticed such a differential recognition of leptospiral lipoproteins between human and mouse TLR2.

Tissue fibrosis is a very complex dynamic process leading to excessive and pathologic accumulation of matrix components that involves many different cells, differentiation and signaling pathways [16]. In a former study, we showed at day-3 p.i. a protective role of T cells, producing IFN-γ and helping macrophages to fight *Leptospira* in the mouse kidney [10]. This finding is in accordance with the earlier study of Martha Pereira who showed that depletion of CD4^+^ and CD8^+^ T cells in mice worsened interstitial nephritis [24]. A role of CD4^+^ T cells in promoting the renal fibrosis has been recently described in a mouse model of renal fibrosis induced by unilateral ureteral obstruction [15]. This model generates a progressive fibrosis in the kidney with interstitial infiltrations of macrophages. The question arises as to whether recruited T cells in kidneys of *Leptospira*-infected mice could, beside their early protective role, have also an adverse effect in promoting the fibrosis at later time points. The use of transgenic mice devoid of T cells (CD3ko mice) showed unambiguously that T cells do not take part of the renal fibrotic process in *Leptospira*-infected mice.

Other cells from the adaptive immunity such as B lymphocytes have been associated with renal fibrotic processes by deposition of immunoglobulins, such as IgG4-related disease, showing high level of serum IgG4 and abundant IgG4-positive plasma cell infiltration into the renal interstitium with fibrosis [33]. We previously showed the crucial and protective role of B cells in leptospirosis through early protective, specific IgM production and later IgG production [10]. The experiment using μMT transgenic mice devoid of B cells and rescued from leptospirosis by administration of immune sera, showed that these mice also exhibit some renal fibrosis, suggesting that B cells and/or related antibody production are not involved in the renal fibrotic process.

We previously showed the important role of both TLR2 and TLR4 in the murine innate defense against *Leptospira* [10], [32]. Surprisingly, we did not find any role of TLR2 nor TLR4 in the induction of renal fibrosis, although recent data indicate a role of TLRs in renal pathologies [34]. Hence, TLR4 activation has been shown to favor kidney fibrosis in the mouse model of unilateral urinary obstruction [14]. TLR2 has also been involved in renal fibrosis after unilateral ureteral obstruction [35], and has been suggested to be important for *Leptospira*-induced fibrosis [9]. However, our *in vivo* results showed that renal fibrosis is still present in TLR2/4 double deficient mice, excluding any major contribution of these receptors, and confirmed the fact that TLR2 agonists such as LipL32 are not major players in triggering renal interstitial fibrosis. Moreover, the fact that *Leptospira*-infected kidneys from MyD88ko and TLR3ko mice were also fibrotic, excludes any important role for TLRs in the mechanism of *Leptospira*-induced renal fibrosis. These rather unexpected results are in accordance with the recent work of Anders's group showing that post obstructive renal fibrosis is independent of TLR2, TLR9 and MyD88 [36].

Apart from the trans-membrane TLR innate immune receptors, the cytosolic family of Nod-like receptors also sense cellular intrusion of pathogens and danger signals. For example, Nod1 and Nod2 detect distinct muropeptides of bacterial peptidoglycan [37]. *Leptospira* species have a peptidoglycan whose chemical composition is close to the one of Gram-negative bacteria despite some peculiarities in their muropeptide composition [38]. However, infected kidneys from Nod1/2dko mice exhibited interstitial fibrosis, therefore excluding a role for both Nod1 and Nod2 in the induction of renal fibrosis. On the other hand, the inflammasome receptor NLRP3, shown to participate in the lung fibrosis induced by uric acid [12], is activated in kidneys from day-3 p.i. with *Leptospira* [11]. We also reported that the activation of the NLRP3 inflammasome does not occur through reactive oxygen species production, but rather more through the effect of the glycolipoprotein, an outer membrane toxin of *Leptospira* inhibiting the sodium/potassium pump (Na/KATPase) in macrophages [11]. This leads to a potassium dysregulation and activation of NLRP3. NLRP3, like most other NLRPs, uses the adaptor ASC to activate caspase1 that in turn cleaves pro-IL1ß, allowing for the IL1ß secretion. Although drugs inhibiting Na/KATPase together with ROS production have been shown to promote renal fibrosis [39], we neither observed any decrease in fibrosis in Casp1ko mice nor in ASCko mice (not shown). Therefore, our results also strikingly exclude a role for NLRs in the *Leptospira*-induced fibrosis.

We previously reported that *Leptospira* induce up-regulation of the iNOS mRNA in kidneys at day-3 p.i. [10]. Here we confirmed the production of NO upon stimulation of bone marrow macrophages with live or dead *Leptospira*. NO production is a potent innate mechanism to eliminate invading bacteria, but upregulation of renal iNOS has also been linked to kidney injury during systemic inflammation [40]. The fact that iNOS deficient mice and antibiotic-treated WT mice were less fibrotic at day-15 p.i., suggests that early iNOS functions, in response to the initial phase of colonization of the kidneys by *Leptospira*, would be important for the fibrogenesis process. However, fibrosis is not abolished in the infected iNOSko mice, suggesting that other unknown mechanisms exist, promoting leptospiral-induced renal fibrosis. iNOS has recently been shown by others to participate in *Leptospira*-induced interstitial nephritis, as measured one month p.i. by a lower histopathological scoring of inflammation in kidneys from iNOSko mice, that were slightly more infected compared to WT mice [5]. Here, we also found a slightly greater number of *Leptospira* in the urine of iNOSko mice, suggesting that NO participates in bacterial clearance. However, we did not find less inflammation in iNOSko mice, although we found less fibrosis, both by scoring and morphometry of Red Sirius, and, down-regulation of fibrosis markers. Although we demonstrated in a previous study that at day-3 p.i. the up-regulation of iNOS is decreased in TLR2/4dko mice [10], TLR2/4dko mice still developed renal fibrosis. One explanation for this apparent discrepancy could be that NO, which we previously found to be released from the parenchymal compartment at day 3 p.i. [10], would be produced by macrophages at later time points through other unknown TLR-independent pathway(s).

Analysis of serum creatinine levels, as a marker of renal function, reveals that WT mice do not show elevated levels of serum creatinine 3 months p.i., confirming their asymptomatic carrier status. However, TLR deficient mice, which harbored more bacteria in their urine, exhibited discretely increased levels of serum creatinine, suggesting that the renal function is slightly affected. Moreover, the renal inflammation in WT mice 6 months p.i., suggests that chronic carriage of *Leptospira* in the long-term could be deleterious. Interestingly, since humans do not sense leptospiral LPS through TLR4 [6], we may hypothesize that chronic carriage of *Leptospira*, already demonstrated in humans [3], [41], [42], could also be linked to a slightly impaired renal function further favoring development of other kidney diseases as previously suggested by Yang's group in Taiwan [4].

To summarize, the present study provides lines of evidences that renal colonization by *Leptospira* induces a mild renal fibrosis in mice through TLR- and NLR-independent pathways, and suggests that the activation of iNOS plays a role in the induction of the renal fibrosis. Our work also highlights the fact that future therapeutic strategies should aim at eliminating *Leptospira* very early after infection, before the renal colonization. Therefore, development of efficient human vaccines against pathogenic *Leptospira* would be extremely useful to prevent chronic carriage of *Leptospira* that may, in the long term, alter renal function.
