# Correction: *Leptospira Interrogans* Induces Fibrosis in the Mouse Kidney through Inos-Dependent, TLR- and NLR-Independent Signaling Pathways

**DOI:** 10.1371/journal.pntd.0002802

**Published:** 2014-04-18

**Authors:** The PLOS Neglected Tropical Diseases Staff

In the author list, it should be indicated that Jean-Michel Goujon and Catherine Werts contributed equally to this work.
